# Virologic Outcome of Using Tenofovir/Emtricitabine to Treat Hepatitis B in HIV-Coinfected Patients

**DOI:** 10.5402/2011/405390

**Published:** 2011-06-13

**Authors:** Christian A. Engell, Vinh Philip Pham, Robert S. Holzman, Judith A. Aberg

**Affiliations:** ^1^Department of Medicine, Division of Infectious Diseases and Immunology, New York University School of Medicine at Bellevue Hospital Center, New York, NY 10016, USA; ^2^Division of Infectious Diseases, Newark Beth Israel Medical Center, 201 Lyons Avenue, Newark, NJ 07112, USA

## Abstract

*Goal*. To study the effect of combination antiviral therapy with tenofovir and emtricitabine or lamivudine with and without prior monotherapy with lamivudine. *Study*. We reviewed charts of 31 HIV-/HBV-coinfected patients. Twelve 3TC-naïve patients initially received tenofovir plus emtricitabine. Nineteen epivir experienced patients who had previously failed epivir were given tenofovir plus emtricitabine. *Results*. Baseline median HBV DNA was similar in the epivir-naïve (5.8*×*10^7^ copies/mL) and experienced group (7.3*×*10^7^ copies/mL, *P* = .65). The median time to complete suppression of HBV was 466 days in the naïve group and 877 days in the experienced (*P* = .001). After 12 months, 6/10 (60%) naïve patients and 3/14 (21%) experienced patients had HBV DNA below the detectionlimit (*P* = .067). After 24 months, 5/5 (100%) naïve patients and 4/13 (31%) experienced patients had an undetectable HBV DNA level (*P* = .015). *Conclusions*. The median time to suppression of HBV DNA was significantly shorter among treatment naïve patients. There was a significantly greater proportion of naïve patients with suppressed HBV DNA at 24 months. Our results support using initial dual therapy in those with HIV/HBV coinfection.

## 1. Introduction

There are an estimated 400 million people worldwide with chronic hepatitis B virus (HBV) infection [1] and an estimated 36 million people infected with human immunodeficiency virus (HIV) [2]. Due to similar modes of transmission, it is not surprising that coinfection with HBV is common, with estimates of the prevalence of coinfection varying from 4 to 23% [3–11]. In those areas where antiretroviral treatment has succeeded in reducing mortality from acquired immune deficiency syndrome (AIDS) and its associated opportunistic infections, the proportion of mortality due to hepatic disease has increased [12–16]. 

There is no conclusive evidence that coinfection with HBV adversely affects the outcome of HIV treatment. Neither the number of AIDS-defining events nor the CD4+ cell response to antiretroviral treatment was affected by being HBV surface antigen positive (HBsAg+) in the EuroSIDA cohort of 9082 HIV infected patients where 8.7% were HBsAg+ [5]. A similar lack of effect was seen in a cohort of 3180 Danish HIV patients, 6% of whom were HBsAg+ [7], as well as in smaller cohorts in Italy [17], South Africa [18], and Taiwan [9]. The consistency of the data suggests that this lack of effect of HBV coinfection on response to ART is independent of demographic factors.

In contrast, HIV coinfection appears to have a negative effect on the course of chronic HBV infection. HIV coinfection is associated with increased HBV replication and levels of HBV viremia and with impaired immune responses to HBV [19–21]. Patients with coinfection have increased rates of fibrosis [22] and accelerated progression to cirrhosis and hepatic failure, with consequent increases in liver-related mortality [5, 23, 24]. Antiretroviral therapy has been shown to improve outcomes for those with HBV monoinfection [25], and it may reasonably be expected to reduce long-term liver-related mortality in HIV-coinfected patients as well [26, 27]. As a result, the IAS-USA and DHHS both recommend that antiretroviral therapy be offered to those with active HBV infection regardless of CD4 T-cell count [28–30] .

Among those with HBV monoinfection, therapy with lamivudine (3TC) has proven to be effective in both reducing HBV viremia and in reducing rates of progression to cirrhosis and hepatocellular carcinoma compared to placebo [25]. However, HBV resistance to 3TC develops relatively quickly. After four years of therapy, up towards 70% of patients treated with 3TC alone for HBV monoinfection have developed resistance [31, 32]. Resistance may develop even more rapidly in those with HIV/HBV coinfection. Only 47% of patients receiving 3TC had HBV DNA levels less than 2.5 pg/mL after two years [33], and after 4 years as many as 94% may have mutations conferring resistance to 3TC [34]. Persons with HIV/HBV coinfection readily accumulate multiple mutations conferring resistance to 3TC [35] and appear to do so more frequently than those with HBV infection alone [36].

Due to the high risk of selecting resistant HBV, ART regimens containing 3TC as the sole agent with activity against HBV are now considered suboptimal for those with HIV/HBV coinfection. ART regimens containing two reverse transcriptase inhibitors active against HBV (i.e., tenofovir (TDF) combined with either 3TC or emtricitabine (FTC)) are therefore currently recommended as first-line therapy for coinfected patients [3, 21, 29, 37, 38]. 

Despite this recommendation, there are limited data demonstrating the superiority of combination therapy using two agents with activity against HBV. For example, a randomized clinical trial comparing 3TC, TDF, and the combination of TDF/3TC demonstrated the expected inferiority of 3TC monotherapy to TDF/3TC. However, it failed to show a significant difference between TDF monotherapy and TDF/3TC [39]. 

In contrast, a cross-sectional study of three cohorts with a combined total of 122 persons with HIV/HBV coinfection showed that those receiving two agents (TDF with either 3TC or FTC) were less likely to have HBV DNA levels greater than 20,000 IU/mL than those receiving either TDF or 3TC/FTC alone at the time of observation [40]. However, due to the design of the study, it was not possible to examine the treatment-induced changes between the different treatment groups. It is therefore not yet clear whether initial treatment with combination therapy in HIV/HBV coinfection improves outcomes compared to combination therapy after failure of 3TC. 

The goal of the current study was to analyze the HBV-related virologic response to the combination of TDF and FTC in 3TC-naïve patients with HIV/HBV coinfection, compared to 3TC-experienced patients who have experienced treatment failure. We evaluated the frequency of complete HBV DNA suppression after one- and two-years treatment and determined the time from starting treatment to complete suppression of measurable HBV DNA.

## 2. Materials and Methods

### 2.1. Patients

We conducted a retrospective review of the electronic medical records (EMR) of patients with HIV/HBV coinfection at the Bellevue Hospital Center (BHC) in New York City during the period 01/01/2002 to 03/31/2008. Inclusion criteria were documented HIV-/HBV-coinfection, age >18 years, ≥2 measurements of quantitative HBV DNA, detectable HBV DNA at baseline, and provision of care at the BHC Virology Clinic. 31 of 159 HIV-/HBV-coinfected patients met the inclusion criteria. Twelve patients who had not previously received 3TC were given initial therapy with TDF and FTC as part of their anti-HIV regimen (the “3TC naïve” group). Thirty-two other patients were initially started on a 3TC-based anti-HIV regimen. Of these 32, 19 failed to maintain a suppressed HBV viral load as evidenced by detectable HBV DNA levels (the “3TC-experienced” group) and therefore met our inclusion criteria. Eleven had undetectable HBV DNA while receiving 3TC as the only HBV-active agent but were changed to a regimen of TDF/FTC, presumably due to concern for long-term resistance. These patients were not included in our study. Three had received 3TC in the past, but their regimen was stopped before clinical or virologic failure could be determined. These 3 patients were also excluded from the analysis. Institutional Review Board approval was obtained for this retrospective study.

### 2.2. Laboratory Testing

Study baseline (Day 0) was defined as the date that TDF and FTC were begun. Baseline lab values included HBV serologies, HBV DNA PCR (viral load), liver function tests, CD4+ cell count, and HIV-1 RNA PCR (viral load). HBV surface antigen (HBsAg) and HBV surface antibody (HBsAb) were determined on a Siemens Centaur analyzer (chemiluminescence immunoassay protocol). HBV DNA PCR was performed at the discretion of the provider using the Roche COBAS assay; the BHC laboratory reported a linear range of 6 IU/mL to 1.10 × 10^8^ IU/mL. HBe antigen (HBeAg) and antibody (HBeAb) testing was performed by EIA at Quest Diagnostics (Teterboro, NJ, USA). HIV-1 RNA PCR was performed using the Roche Amplicor test (lower limit of detection 50 copies/mL). CD4+ lymphocyte counts were performed at BHC using a Fluorescence Activated Cell Sorter. ALT measurement was performed using a Siemens Advia 2400 analyzer, with an upper limit of normal of 35 IU/L.

### 2.3. Statistical Analysis

Time to undetectable viral load was estimated by Kaplan-Meier analysis with the Log Rank test used to estimate significance. The time at risk was the time from baseline to the last measurement of HBV DNA (for censored observations) or until the first undetectable level of HBV DNA. 

Statistical analyses were performed with SPSS for windows (version 17.0: SPSS inc., Chicago, Ill, USA). The Chi-square test was used to compare categorical variables. The two sided *t*-test for equality of means was used for continuous variables. *P* values of <.05 were deemed statistically significant, and those between.05 and.10 were deemed to suggest a trend in the data. 

## 3. Results

At baseline, the mean age and proportion of male patients were similar amongst the 3TC-naïve and 3TC-experienced patients (Table 1). The 3TC-experienced group had significantly higher CD4+ cell counts (155 versus 333, *P* = .003) and nonsignificantly lower HIV viral loads than the 3TC-naïve group at baseline (5.93 × 10^4^ versus 3.00 × 10^4^, *P* = .17).

The 3TC-naïve group had a median HBV DNA of 5.8 × 10^7^ copies/mL compared with 7.3 × 10^7^ copies/mL in the 3TC experienced (*P* = .60). Although the mean ALT in the 3TC-experienced group was higher than for the 3TC-naïve group (82 versus 46 IU/L), the difference between the groups was not statistically significant (*P* = .10).

**Figure 1:**
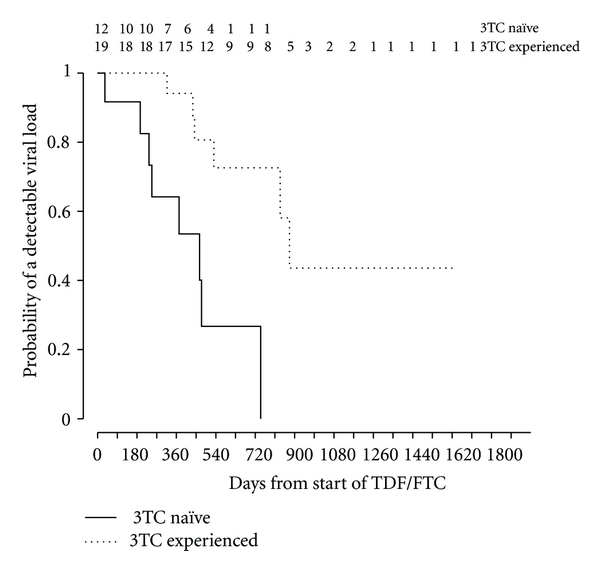
Kaplan-Meier function of time to suppression.

The median time to complete suppression of HBV DNA in the 3TC-naïve patients was 466 days, compared with 877 days in the 3TC-experienced group (*P* = .001) (Figure 1). At the time of HBV DNA suppression or last recorded HBV DNA level, there was no significant difference between the two groups in HIV RNA suppression (8/12 versus 13/19, *P* = .92).

After 12 months, 6/10 (60%) 3TC-naïve patients but only 3/14 (21%) 3TC-experienced patients had an undetectable HBV DNA (*P* = .092) (Table 2). After 24 months, 5/5 (100%) of 3TC-naïve patients but only 4/13 (31%) 3TC-experienced patients had an undetectable HBV DNA (*P* = .015) (Table 3). Among those who were initially HBeAg+, loss of detectable HBeAg occurred in 1/7 (14%) 3TC-naïve and 1/11 (9%) 3TC-experienced patients.

## 4. Discussion

The present study shows that for persons coinfected with HIV and HBV, initial treatment with a combination of two agents that have activity against HBV resulted in a significantly shorter time to suppression of HBV DNA level, as compared to using combination therapy after prior monotherapy with 3TC. Our study supplements a prior retrospective study which compared 10 persons who received TDF + 3TC as part of their initial regimen with 20 3TC-experienced persons who received TDF add-on; in that study, a greater proportion of persons initially receiving combination therapy achieved HBV DNA <2000 copies/mL (80% versus 55%) at one year of followup, although the result did not reach statistical significance [41].

These observations are in contrast to those of two other studies. In one retrospective study, 25 treatment-naïve patients who received TDF + 3TC were compared with 50 3TC-experienced patients who experienced HBV virologic breakthrough and then had TDF added; of those who initially received combination therapy, 76% achieved HBV DNA levels <1000 copies/mL, compared with 84% who had TDF added after 3TC failure, a difference that was not statistically significant [42]. Similarly, in another retrospective study of 52 mostly 3TC-experienced patients who subsequently received TDF, all 9 patients with virologic breakthrough were receiving TDF in combination with either 3TC or FTC, whereas none of the 9 patients receiving TDF alone experienced virologic breakthrough [43]. The authors of these studies have concluded that they could not show an advantage to combination therapy within the follow-up period of their studies.

Our report and that of Jain et al. [26] therefore provide some of the first data to support the current guidelines and expert recommendations for the use of two antiviral agents with activity against HBV; in addition, the present study demonstrates an advantage of combination therapy at longer followup than was reported previously. Nevertheless, both studies suggest that initial combination therapy is superior to rescue therapy for persons with HIV/HBV coinfection. While there are clearly methodological differences between these studies, the similarity of the observations provides a basis for further investigation.

A benefit of initial combination therapy against HBV also reinforces the need to assess the baseline HBV status of the patient prior to starting antiviral therapy for HIV. Inappropriate antiviral therapy that only includes one agent active against HBV could lead to long-term consequences regarding HBV suppression, which in turn could ultimately affect risk of liver-related mortality.

The current study has limitations due to its retrospective design. Data on previous length of 3TC therapy and treatment interruptions was incomplete. Data on cirrhosis and liver histology was also incomplete. Monitoring of HBV DNA levels and serologic markers was at the discretion of the individual providers, so there were differences in the intensity of surveillance of viral loads among the 31 patients. Also, the study population was small, limiting its ability to detect small differences. In spite of this limitation, the median time to HBV virologic suppression was substantially and significantly shorter in the 3TC-naïve group. There was also greater suppression of HBV DNA at 24 months in 3TC-naïve patients, and the difference again was statistically significant despite the small study size. 

Differences in baseline status are potential confounders in retrospective studies. For example, it has been noted that greater HBV DNA baseline levels may result in the detection of greater HBV DNA decreases on therapy [22]. However, this was unlikely to be a major factor in our study since baseline HBV DNA levels were similar in both our 3TC-naïve and -experienced groups. Similarly, another potential confounder could have been baseline CD4+ cell count. There is evidence that improvement in CD4+ lymphocyte count during ART is associated with improvement in the evolution of HBV disease [44–46]. Indeed, HBV-specific CD4+ lymphocyte activity has been shown to be important for control of HBV infection in those without HIV coinfection as well [47–49]. However, in the current study, the mean CD4+ cell count was higher in the 3TC-experienced group, suggesting that the observed superiority of combination therapy against HBV in the 3TC-naïve group was unlikely to be the result of better immunologic status in that group.

A difference in adherence to treatment is another potential confounder in retrospective studies, and we did not directly analyze provider documented adherence rates. If the 3TC-experienced group included more persons with nonadherence to their ART regimen, then 3TC failure due to nonadherence may increase TDF/FTC failure due to nonadherence to TDF/FTC as well and bias the results against the 3TC-exprienced group. However, adherence may be inferred by assessing control of HIV viremia; since HIV viremia on ART did not differ between the groups, we believe that any differences in adherence would have had, at most, a limited effect on the results.

In summary, our findings show that patients who are initially started on dual therapy with tenofovir and emtricitabine have a shorter median time to suppression of HBV DNA than those who are treated with tenofovir and emtricitabine after prior treatment with lamivudine; this observation suggests a benefit from initially treating all persons with HIV/HBV coinfection with ART containing two agents with activity against HBV. Our findings therefore support the current practice of starting dual therapy for hepatitis B in HIV-/HBV-coinfected patients. Further studies are needed to understand the clinical consequence of this, and whether time to suppression will result in improved clinical outcomes regarding liver-related mortality. In addition to providing evidence to support current treatment recommendations for HIV/HBV coinfection, we speculate that such studies may also eventually warrant investigation of potential benefit for those with HBV monoinfection.

##  Disclosure

C. A. Engell and R. S. Holzman declare that there is no conflict of interests.Vinh Pham has served on Gilead Advisory Board Judith A Aberg has served as consultant and has been a local investigator on multicentered trials for Bristol-Myers-Squibb, Gilead and Glaxo-Smith-Kline.

## Figures and Tables

**Table 1:** Baseline characteristics of 3TC-naïve and experienced groups.

	3TC naïve (*n* = 12)	3TC experienced (*n* = 19)	
Mean Age	46	45	*P* = .89
Male, % of patients	83%	89%	*P* = .54
Mean HBV DNA level (IU/mL)	5.8 × 10^7^	7.6 × 10^7^	*P* = .60
Mean ALT level (IU/L)	46	82	*P* = .10
Mean CD4 count (cells/mm^3^)	155	333	*P* = .003
Mean HIV RNA level (Copies/mL)	59,285	30,034	*P* = .17
HBeAg positive, no. of pts (%)	7 (58%)	11 (69%)	*P* = .728
HCV infection	1	0	

**Table 2:** Patients with suppressed HBV VL 12 months after starting TDF/FTC.

	Detectable	Suppressed	*P* = .067
3TC naïve (*n* = 10)	4 (40%)	6 (60%)	10 (100%)
3TC experienced (*n* = 14)	11 (79%)	3 (21%)	14 (100%)

Total	15	9	24

**Table 3:** Patients with suppressed HBV VL 24 months after starting TDF/FTC.

	Detectable	Suppressed	*P* = .015
3TC naïve (*n* = 5)	0 (0%)	5 (100%)	5 (100%)
3TC experienced (*n* = 13)	9 (69%)	4 (31%)	13 (100%)

Total	9	9	18
